# Evaluation of Extraction Methods for Clinical Metagenomic Assay

**DOI:** 10.3390/microorganisms8081128

**Published:** 2020-07-27

**Authors:** Suha A. Farraj, Shreif A. El-Kafrawy, Taha A. Kumosani, Jehad M. Yousef, Esam I. Azhar

**Affiliations:** 1 Biochemistry Department, Faculty of Sciences, King Abdulaziz University, Jeddah 21589, Saudi Arabia; sfaraj0008@stu.kau.edu.sa (S.A.F.); tkumosani@kau.edu.sa (T.A.K.); jyousef@kau.edu.sa (J.M.Y.); 2 Special Infectious Agents Unit, King Fahd Medical Research Center, King Abdulaziz University, Jeddah 21589, Saudi Arabia; saelkfrawy@kau.edu.sa; 3 Clinical Pathology Department, National Liver Institute, Menoufia University, Shebin El-Kom 32511, Egypt; 4 Medical Laboratory Technology Department, Faculty of Applied Medical Sciences, King Abdulaziz University, Jeddah 21589, Saudi Arabia; 5 Central Laboratory for Food and Nutrition, King Abdulaziz University, Jeddah 21589, Saudi Arabia; 6 Department of Biochemistry, College of Sciences, University of Jeddah, Jeddah 23890, Saudi Arabia

**Keywords:** clinical metagenomics, nucleic acid extraction, microorganisms, infectious diseases

## Abstract

(1) Background: Clinical metagenomics is a promising approach that helps to identify etiological agents in cases of unknown infections. For the efficient detection of an unknown pathogen, the extraction method must be carefully selected for the maximum recovery of nucleic acid from different microorganisms. The aim of this study was to evaluate different extraction methods that have the ability to isolate nucleic acids from different types of pathogens with good quality and quantity for efficient use in clinical metagenomic identification. (2) Methods: A mock sample spiked with five different pathogens was used for the comparative evaluation of different commercial extraction kits. Extracted samples were subjected to library preparation and run on MiSeq. The selected extraction method based on the outcome of the comparative evaluation was used subsequently for the nucleic acid isolation of all infectious agents in clinical respiratory samples with multiple infections. (3) Results: The protocol using the PowerViral^®^ Environmental RNA-DNA Isolation Kit with a 5-min bead beating step achieved the best results with a low starting volume. The analysis of the tested clinical specimens showed the ability to successfully identify different types of pathogens. (4) Conclusions: The optimized extraction protocol in this study is recommended for clinical metagenomics application in specimens with multiple infections from different taxa.

## 1. Introduction

Globally, infectious diseases are still the leading cause of human morbidity and mortality [1]. Respiratory infections are considered as the third leading cause of death worldwide and the leading cause of death in developed countries, resulting in nearly 4.18 million deaths per year [2,3]. The main limitation to prevent and minimize the burden of infectious disease is to establish a rapid and accurate laboratory method with the ability to identify the etiological agent associated with the infection [4]. Throughout observations in the clinical setting, a significant number of infectious diseases have been unidentifiable using currently available laboratory tests [5]. Approximately 30–70% of the pathogens associated with pneumonia, meningitis, and encephalitis are routinely unidentified in clinical laboratories [2,3,6]. The limited identification of a wide range of infectious agents that are associated with human infections using currently available methods makes it really difficult to diagnose diseases, which highly affects the clinical management of such cases and might lead to potentially adverse reactions due to improper treatment [7]. Thus, next-generation sequencing (NGS) may provide the solution for such problem as it has the ability to identify almost all microorganisms in a clinical sample without prior knowledge of the target [8], allowing the identification of known and novel infectious agents in different human specimens regardless of the organism type associated with the infection. Furthermore, NGS allows clinical laboratory scientists to identify single or multiple infections simultaneously [1,9]. In essence, NGS can be utilized for cases in the field known as clinical metagenomics (CMg), which expected to become the leading diagnostic method in the near future for the identification of pathogens associated with infection, especially during outbreak investigations [5,10]. 

However, clinical scientists still face difficulties in applying CMg because it requires different NGS processes to be optimized for pathogen detection purposes [10]. The guarantee of pathogen recovery through the efficient extraction of diverse microbial species is a challenging issue [10,11]. A number of kits are commercially available for DNA and RNA extraction. However, some microorganisms, such as fungi, have a rigid wall membrane that requires additional modification processes to isolate their nucleic acid [12]. Conversely, the modification process must be carefully considered because it may lead to viral nucleic acid degradation [10,11]. Thus, the main aim of this study was to compare the outcomes of different extraction methods and optimize the protocol of choice.

## 2. Materials and Methods 

All the experiments involved in this study were performed at the Special Infectious Agents Unit (SIAU), King Fahd Medical Research Center (KFMRC), King Abdulaziz University (KAU), Jeddah, Saudi Arabia.

### 2.1. Mock Sample Preparation

The mock sample was composed of equal volumes of different well identified and characterized infectious agents currently available at SIAU or obtained from different reference institutes as stated below. The mock sample used in this study includes the following microorganisms; two viruses isolated at SIAU with a full genome characterization [13], adenovirus (AdV), a nonenveloped double-stranded DNA virus, isolated in HeLa cells with a virus titer of 4.2 × 10^5^ fifty-percent tissue culture infective dose/mL (TCID50/mL); and Alkhumrah virus (ALKV), an enveloped single-stranded RNA virus, isolated in Vero cells with a virus titer of 5.6 × 10^5^ TCID50. *Staphylococcus aureus* (*S. aureus*) “National Collection of Type Cultures (NCTC)# 8325” and *Klebsiella pneumoniae* (*K. pneumoniae*), isolated and characterized from a clinical sample at the clinical microbiology lab of King Abdulaziz University Hospital (KAUH), were chosen to represent Gram-positive bacteria and Gram-negative bacteria, respectively, with colony-forming units (cfu) of 8.6 × 10^7^ cfu/mL for *S. aureus* and 9.4 × 10^6^ cfu/mL for *K. pneumoniae*. Finally, the encapsulated yeast *Cryptococcus neoformans* (*C. neoformans*) with a cfu of 1.05 × 10^6^ cfu/mL was isolated and characterized from a clinical sample at the mycology lab of KAUH. 

### 2.2. Extraction Protocols

**Figure 1:**
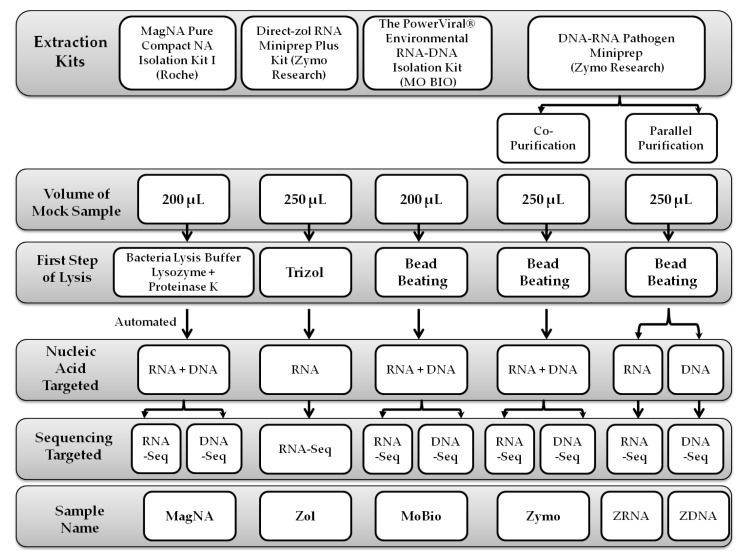
Schematic presentation of the extraction protocols used for comparison in this study. DNA -Seq stand for DNA sequencing and RNA-Seq stand for RNA sequencing.

This study compared four different commercial extraction kits, where one of them performed in three different protocols to be able to isolate the nucleic acid in different forms as RNA and DNA, and both are based on the manufacturers’ instructions as shown in Figure 1; (1) the MagNA Pure Compact NA Isolation Kit I (Roche, Penzberg, Germany); (2) the Direct-zol RNA Miniprep Plus Kit (Zymo Research, Irvine, CA, USA); (3) the PowerViral^®^ Environmental RNA-DNA Isolation Kit (MO BIO Laboratories, Carlsbad, CA, USA), which is currently sold under the name “Allprep_PowerViral_DNARNA” (Qiagen, Hilden, Germany); and (4) the DNA-RNA Pathogen Miniprep (Zymo Research Irvine, CA, USA). Regardless of the volume of the starting material, the extracted samples were eluted in 50 µL of DNase-/RNase-free water, and each protocol was evaluated using two independent samples.

#### 2.2.1. MagNA Pure Compact NA Isolation Protocol

This protocol yielded total nucleic acids and was the only automated extraction method in this study that used the MagNA Pure Compact Instrument (Roche Diagnostics, Mannheim, Germany), which is a magnetic bead-based technology. Briefly, a pretreatment step was performed using MagNA Pure Bacteria Lysis Buffer (BLB) (Roche Diagnostics, Mannheim, Germany) following the product instructions with slight modifications to ensure complete lysis of the bacteria. First, 200 µL of the mock sample was mixed with 180 µL of BLB. Then, 10 µL of 100 mg/mL lysozyme was added, and the mixture was incubated at 37 °C for 30 min in a ThermoMixer (Eppendorf, Hamburg, Germany) with mixing at 700 rpm. After incubation, 20 µL of 10 mg/mL proteinase K was added and incubated at 65 °C for 10 min. The treated samples were cooled on ice and then extracted with the MagNA Pure Compact NA Isolation Kit I. The eluted samples were labeled “MagNA”. 

#### 2.2.2. Direct-zol™ RNA Miniprep Plus Protocol

This protocol yielded RNA only. The mock sample (250 µL) was mixed with 750 µL of TRIzol™ LS Reagent (Ambion Life Technologies, Carlsbad, CA, USA) as per the manufacturer’s instruction. Briefly, the mixture was applied directly to the provided column from the Direct-zol™ RNA Miniprep Plus Kit without phase separation. Subsequently, the lysate was subjected to on-column DNA digestion according to the protocol. The eluted samples were labeled as “Zol”.

#### 2.2.3. PowerViral^®^ Environmental RNA/DNA Isolation Protocol

This protocol was intended for total nucleic acids extraction using the PowerViral^®^ Environmental RNA/DNA Isolation Kit, which is a filter-based technique. The manufacturer’s instructions were followed with a slight modification. Briefly, 200 µL of the mock sample was mixed with 600 µL of prewarmed PV1 buffer and 6 µL of β-mercaptoethanol (Sigma, Heidelberg, Germany). The mixture was added to ZR BashingBead Lysis Tubes with mixed sizes (0.5 mm and 0.1 mm) of beads (Zymo Research, Irvine, USA). The tube was placed in a TissueLyser (Qiagen, Hilden, Germany) for 25 s, followed by a 5-s break and another 25 s of agitation at 30 Hz. The resulting mixture was centrifuged at 4 °C (5430 R; Eppendorf, Hamburg, Germany), and the supernatant was obtained by following the kit instructions. The eluted samples were labeled “MoBio”. 

#### 2.2.4. ZymoBIOMICS™ DNA/RNA Miniprep Protocols

Three protocols were performed using the ZymoBIOMICS™ DNA/RNA Miniprep Kit, which is a filter-based technique with two different procedures: “Copurification”, which yields total nucleic acids, and “Parallel Purification”, in which DNA and RNA are eluted separately. 

As a common step for both procedures, the beating step was performed as described in the previous protocol for a mixture consisting of 250 µL of the mock sample with 750 µL of DNA/RNA Shield. Then, the protocol in the instructions was followed, and nucleic acids that were eluted in the “Parallel Purification” protocol were labeled “ZDNA” for the DNA samples and “ZRNA” for the parallel RNA samples, while nucleic acids that were eluted in the “Copurification” protocol were labeled “Zymo”. 

### 2.3. Nucleic Acid Quantification

Extracted nucleic acids were quantified by Qubit 2.0 (Invitrogen Life technologies, Carlsbad, CA, USA) using both the Qubit™ dsDNA HS Assay Kit and Qubit™ RNA HS Assay Kit (Life Technologies, Eugene, OR, USA) following the manufacturer’s instructions. 

### 2.4. Library Preparation

The library preparation kit was selected according to the target nucleic acids, as described in the subsequent sections.

#### 2.4.1. Preparation of the RNA Library

The manufacturer’s instructions of the KAPA RNA HyperPrep Kit for Illumina sequencing (KABA Biosystems, Cape Town, South Africa) were followed to prepare libraries from the RNA extracted after diluting the samples, if needed, to a concentration of 100 ng in 10 µL using elution buffer EB (Qiagen, Hilden, Germany). 

#### 2.4.2. Preparation of the DNA Library 

For DNA-Seq, the Nextera DNA Flex Library Prep (Illumina, San Diego, CA, USA) was prepared according to the manufacturer’s instructions for DNA libraries, with at least 10 µL of each sample with concentrations ranging between 50 and 500 ng, as per the kit recommendation. 

### 2.5. MiSeq Sequencing

In both library preparation types, the concentration of each library was quantified using the Qubit™ dsDNA HS Assay Kit, and the average library size was estimated by the 2100 Bioanalyzer System (Agilent, Waldbronn, Germany) with an Agilent High Sensitivity DNA Kit (Agilent, Santa Clara, CA, USA). The final pooled library was loaded using the 300 cycles MiSeq Reagent Kit v2 (Illumina, Singapore), and the run was performed on a MiSeqDX platform (Illumina, San Diego, CA, USA) to generate paired-end reads.

### 2.6. Analysis of NGS Data

The analysis was performed using the GENEIOUS Prime software (2019.1., Biomatters Ltd., Auckland, New Zealand). First, paired reads were merged together, and duplicates were removed using the Dedupe algorithm. The poor-quality sequences from both ends were trimmed with the BBDuk algorithm with an error probability = 0.05. A reference for each organism in the mock sample (Table 1) was loaded, each sample was mapped using the standard GENEIOUS mapper sequentially against *C. neoformans*, and then the unused reads were mapped to *K. pneumoniae*, *S. aureus*, AdV, and, finally, ALKV. The NGS yields of the extraction assays were compared in two ways: (1) comparison of the number of reads mapped to the reference by calculating the reads per million (RPM) following the equation (No. of Reads Mapped/Total No. of Reads w/o duplicates) × 10^6^; (2) comparison of the coverage percentage (coverage %) of each microorganism reference. 

### 2.7. Bead Beating Optimization

The bead beating technique was involved in a number of the protocols used in this study. Optimization of the bead beating step was performed to improve the yield of the difficult-to-lyse organisms, such as fungi and some Gram-positive bacteria. Because the samples could contain RNA viruses, which are easily degraded by the heat generated from the beating process, the process was performed in a cold room (4°C). After testing different bead beating cycles by real-time PCR with both *C. neoformans* and ALKV (see Appendix A), bead beating continuously for 5 min was chosen for subsequent analysis with NGS using PowerViral^®^ Environmental RNA/DNA Isolation protocol (5-min MoBio samples) and ZymoBIOMICS™ DNA/RNA Miniprep copurification protocol (5-min Zymo sample). 

### 2.8. Statistical Analysis

Using SPSS Statistics (Subscriptions, IBM, Armonk, New York, United States), one-way ANOVA followed by Bonferroni’s post hoc test was used to check the statistical significance of the differences between assays, and the *p* value was considered statistically significant if it was equal to or less than 0.05. 

### 2.9. Clinical Samples

Throat swabs were collected from patients admitted to KAUH and routinely submitted to SIAU for diagnosis against a panel of respiratory pathogens. The samples that tested positive for one or more respiratory pathogens were selected for the validation of the optimized extraction protocol under the ethical approval number 290-17, dated 13 June 2017, from the Unit of Biomedical Ethics, King Abdulaziz University Hospital.

### 2.10. Analysis of Clinical Metagenomics 

An establishment protocol (see Appendix B) was followed that targeted all microbes in a sample, disregarding their taxa or genome type. 

The generated reads were analyzed using CosmosID’s bioinformatics platform online app (https://www.cosmosid.com/platform) (1.0, Rockville, MD, USA) against its databases of bacteria, viruses, fungi, and protists by identifying unique and shared k-mers in the reference genome and searching for a match in the queried metagenomic sample. By using the filtration property, which depends on internal statistical scores, the confirmed organisms that were likely to be in the sample and the unconfirmed organisms needed to be validated by another laboratory test. The frequency (f), which is the number of unique k-mers found in the queried sample belonging to a referenced organism, was used for analysis. 

## 3. Results

### 3.1. Sequencing RNA Targets

RNA was extracted separately or combined with DNA from the mock sample by five different protocols: (1) the automated assay using MagNA Pure (MagNa samples); (2) Direct-zol RNA Miniprep Plus (Zol samples); (3) the PowerViral^®^ Environmental RNA-DNA Isolation Kit (MoBio samples); (4) the ZymoBIOMICS DNA-RNA Miniprep “Copurification” protocol (Zymo samples); and (5) the ZymoBIOMICS DNA-RNA Miniprep “Parallel Purification” protocol (ZRNA samples).

The highest RNA concentration was found with the extraction protocols that did not use bead beating. MagNA pure produced the highest RNA yield (32.75 ng/µL), followed by Zol (18.65 ng/µL). The Zymo and ZRNA samples, which were both extracted using the same kit with different protocols, provided approximately the same yield, with an average of 12.5 ng/µL. Despite the use of bead beating in the MoBio samples, as in the case of the Zymo and ZRNA samples, the sample had a lower concentration of RNA than the detection limit (<20 ng/mL). According to the KAPA RNA HyperPrep protocol, 10 µL was used directly without any dilution of the MoBio samples, and 14 cycles were used in the amplification step instead of the 6 cycles used for the other samples with higher concentrations. 

The average number of reads generated for each sample after merging the R1 and R2 reads was 3,822,832, ranging from 6,387,496 for the MoBio samples to 2,741,110 for the MagNA samples. After removing duplicates, the reads decreased by approximately 1% in the Zol and ZRNA samples, 7% in the MagNA and MoBio samples, and 20% in the Zymo samples.

**Figure A1:**
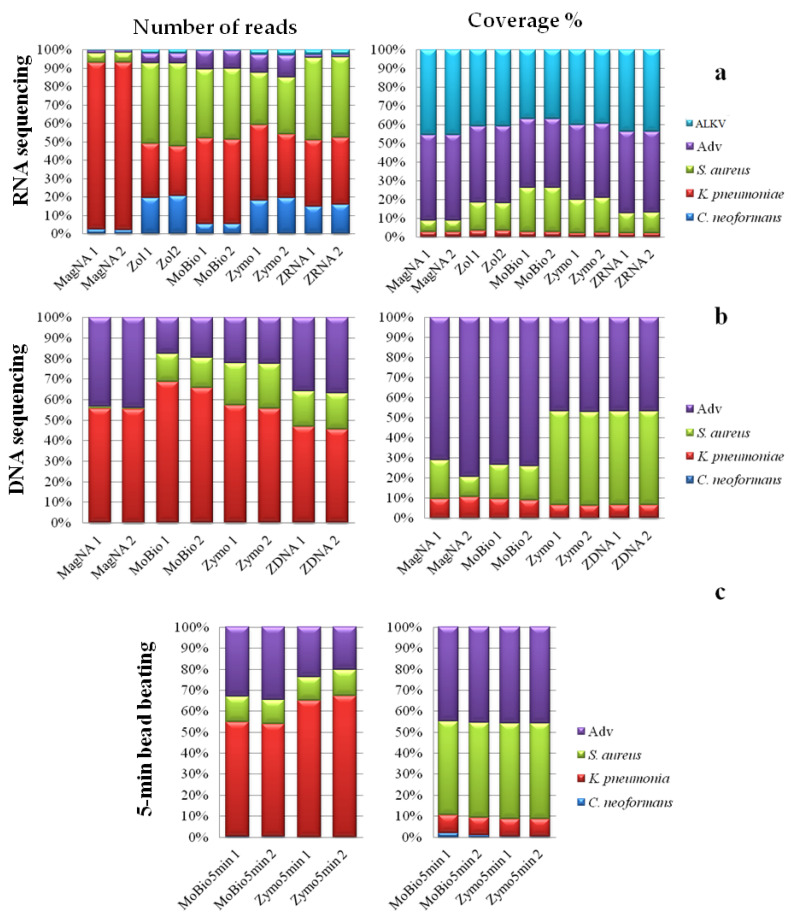
Reads and coverage of microbes in the mock sample. The diagrams on the left represent the number of reads assembled for the spiked pathogens, and those on the right show the percentage of the microbe reference covered by the reads for the duplicates of each extraction method. (**a**) Generated data from RNA sequencing; (**b**) generated data from DNA sequencing; and (**c**) generated data from 5-min bead beating agitation.

For comparison between the protocols, Figure A1a in the Appendix C shows the reads and reference coverage of spiked pathogens between the duplicates of RNA extraction protocols. Table 2 and Table 3 show the average of the mapped reads and reference coverage, respectively, for each spiked pathogen in all extraction protocols. 

For *C. neoformans*, Zymo and Zol had the highest RPM (90,684 RPM and 82,441 RPM, respectively), with no significant difference between them (*p* = 0.91), whereas significance (*p* ≤ 0.001) was found when compared with MoBio (30,427 RPM) and MagNA (19,257 RPM). The highest coverage % was obtained for Zymo and ZRNA at 0.09% and 0.08%, respectively, with no significant difference between them or compared with any other methods (*p* ≥ 0.26).

For *K. pneumoniae*, the MagNA sample showed the highest RPM (784,302 RPM), with *p* ≤ 0.001 compared with other methods. The highest reference coverage for *K. pneumoniae* was obtained by Zol (7.69%), followed by the MoBio coverage (7.20%), but with no significant difference (*p* = 0.30), whereas significance was found when compared with the rest of the extraction methods (*p* ≤ 0.05). 

For *S. aureus*, ZRNA and MoBio showed the highest RPM (216,660 and 210,541, respectively), with no significant difference (*p* = 1.00), but significance was found when compared with both MagNA (46,263 RPM) and Zymo (143,569), with *p* value ≤ 0.024. For the reference coverage, MoBio had the highest coverage (64.10%), with a significant difference (*p* ≤ 0.001) compared with the rest of the extraction methods. 

For AdV, the highest numbers of reads were obtained for MoBio (54,898 RPM) and Zymo (53,243 RPM); the difference between them was not significant (*p* = 1.00), but significant differences were found when compared with the others (*p* value ≤ 0.001). For ALKV, ZRNA and Zymo had the highest numbers of reads (12,945 and 12,830, respectively), with no significant difference between them (*p* = 1.00); however, differences were significant when compared with the others (*p* value ≤ 0.039). In all the extraction assays, the genomes of both Adv and ALKV were ≈100% covered regardless of the difference in RPM. 

### 3.2. Sequencing DNA Targets

DNA was extracted from the mock sample using four different protocols: (1) the automated assay using MagNA Pure (MagNa samples); (2) the PowerViral^®^ Environmental RNA-DNA Isolation (MoBio samples); (3) the ZymoBIOMICS DNA-RNA Miniprep “Copurification” protocol (Zymo samples); and (4) the ZymoBIOMICS DNA-RNA Miniprep “Parallel Purification” protocol (ZDNA samples).

The highest DNA concentration was again obtained using the MagNA samples (14.1 ng/µL). The ZDNA samples had a higher concentration than that of the Zymo samples (13 ng/µL vs. 9.2 ng/µL), which were both extracted using the same kit with different protocols. The concentration was the lowest in the MoBio samples (3.3 ng/µL), as observed from its RNA concentration. For this, 17 µL of the MoBio samples were used for library preparation instead of the 10 µL used in the other DNA extraction protocols.

On average, the number of merged paired reads of each sample was 3,920,271, with the maximum number of reads (6,140,864 reads) obtained for ZDNA samples and the minimum number of reads (140,716 reads) for MoBio samples. The duplicate reads did not exceed 0.2% of the generated reads in all protocols. 

MoBio had the highest RPM for three out of four DNA pathogens in the mock sample: *C. neoformans* (601 RPM), with a significant difference (*p* ≤ 0.001) compared with the other methods; while *S. aureus* (40,813 RPM), shown a significant difference (*p* ≤ 0.002) in contrast with the other methods; and *K. pneumoniae* (192,812 RPM), with no significant difference compared with the next highest result, i.e., MagNa (152,023 RPM) with *p* = 0.07, but with a significant difference compared with the other protocols (*p* ≤ 0.004). Finally, for Adv, MagNa had the highest RPM (119,851 RPM), with a statistically significant difference (*p* ≤ 0.001) compared with the other methods (Table 4, Figure A1b in the Appendix C).

For the reference coverage %, ZDNA had the highest percentage for *C. neoformans* (0.48%), with a significant difference (*p* ≤ 0.026) over the other methods. For *K. pneumoniae,* MagNA, Zymo, and ZDNA showed the highest coverage %, with 13.51%, followed by MoBio, with 12.41%, with no significant differences between any of the extraction protocols (*p* ≥ 0.30). For *S. aureus*, Zymo and ZDNA covered almost the whole reference sequence (~99.9%), with a significant difference (*p* = 0.001) compared with MoBio, which covered only 23%. For Adv, all the extracted assays covered 100% of the Adv genome. As expected, no reads covered ALKV because it is an RNA virus (Table 5, Figure A1b in the Appendix C).

### 3.3. Bead Beating Optimization 

Because the extraction protocols that depend on bead beating in their lysis step showed interesting results in DNA and RNA extraction, an attempt to improve their NGS results, especially with *C. neoformans*, was made. Bead beating with an increasing interval time with or without 5-s breaks using a TissueLyser was performed in a cold room (4 °C).

The protocol with 5 min of continuous bead beating provided the best results when assessed by real-time PCR (see Appendix B). The same mock sample (*C. neoformans*, *K. pneumoniae*, *S. aureus*, AdV and ALKV) was extracted with MoBio and Zymo protocols with a 5-min bead beating step and subjected to DNA-Seq (see Figure A1c in the Appendix C). 

**Figure 2:**
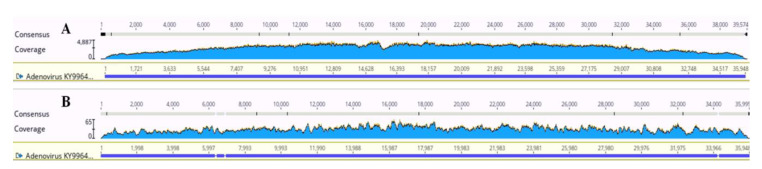
The depth coverage of AdV for the MoBio samples. (**A**) The sample with 5 min of bead beating in the lysis step of extraction. (**B**) The same sample with 25 s × 2 cycles of bead beating.

Comparing the results obtained from 5-min bead beating revealed an improvement in both the number of reads and reference coverage % for all DNA pathogens compared with the previous results using 2 cycles of 25 s. For the 5-min MoBio samples, significant increases were found in the reads of *C. neoformans* (*p* = 0.024) and AdV (*p* = 0.002) and in the reference coverages of *K. pneumoniae* (*p* = 0.001) and *S. aureus* (*p* ≤ 0.001). For the 5-min Zymo samples, a significant increase in the mapped reads was observed for Adv (*p* = 0.05) and *K. pneumoniae* (*p* = 0.004) and in the reference coverage for *K. pneumoniae* (*p* = 0.002). Consequently, the coverage that was already 100% with 25 s × 2 cycles was deepened with 5 min of bead beating. For AdV in the MoBio samples, the mean depth was 30 with 25 s × 2 cycles and became 1828 with 5 min of bead beating (Figure 2). 

In a comparison between the 5-min bead beating results of the two protocols (Table 6), significant increases were found only for 5-min MoBio in the number of reads of *C. neoformans* and AdV, with *p* = 0.01 and 0.008, respectively.

To check the ability of MoBio protocol to extract pathogens from different taxa in real clinical sample, a comparison was done with Zymo protocol where both protocols were applied on a clinical throat swab after 5 min bead beating. The results from MoBio protocol were superior to the Zymo protocol where it was able to detect three extra bacteria genera, which were *Rothia*, *Neisseria*, and *Campylobacter*. The metagenomics results (Table 7) showed the ability of both protocols to detect RNA virus (Human metapneumovirus), DNA viruses (ex. Staphylococcus phages), Gram-negative bacteria (as *Kingella denitrificans* and *Pseudomonas aeruginos*), and Gram-positive bacteria (as *Staphylococcus lugdunensis* and *Staphylococcus aureus)* beside the amoeba Naegleria fowleri. 

### 3.4. Clinical Samples Analysis

Six throat swabs were chosen for clinical metagenomic analysis using the MoBio extraction protocol with 5-min bead beating. One minute of incubation on ice between each minute of beating was added as a precautionary step to avoid RNA degradation for other RNA viruses that were not tested. The generated data were uploaded to the National Center for Biotechnology Information (NCBI) under the Sequence Read Archive (SRA) accession no. PRJNA636773. The generated sequences were submitted to the CosmosID app for analysis. A heat map for the filtered microbes based on frequencies (f) generated from the CosmosID app is shown in Table A3 in the Appendix D.

#### 3.4.1. Sample No. 1

**Figure 3:**
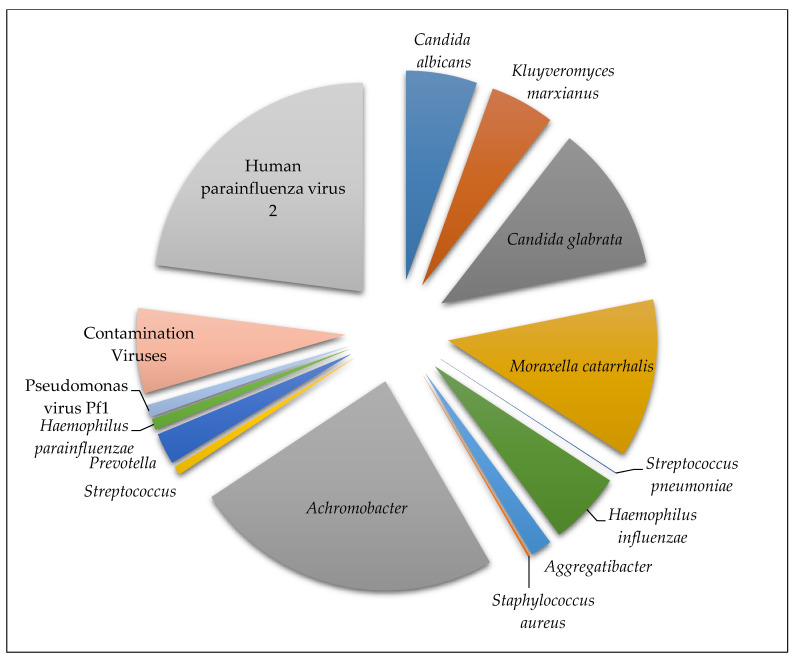
Distribution of microorganisms in sample 1: the filtered microbes plus the confirmed unfiltered pathogens with frequencies generated from the CosmosID app. The group labeled “contamination viruses” comprises viruses found in the negative control.

A 4-year-old male was hospitalized in the pediatric intensive care unit of KAUH because of chronic lung disease with previous multiple admissions due to chest infection aspiration. The analysis of his throat swab sample showed pathogens from different taxonomies. After applying CosmosID filtration, the fungal species *Candida glabrata*, *Candida albicans*, and *Kluyveromyces marxianus* were found in the sample with 799 f, 387 f, and 352 f, respectively. Additionally, Gram-negative bacteria were found, which were identified as *Moraxella catarrhalis* (867 f) and *Haemophilus influenzae* KR494 serotype f (398 f). Finally, the single-stranded RNA virus Human parainfluenza virus 2 was present with 2363 f. Without filtration, both Gram-positive bacteria *Streptococcus pneumoniae* and *Staphylococcus aureus* were detected in the sample with low frequencies (3 f and 10 f, respectively), but their existence was confirmed by the FTD Respiratory Pathogens kit (Figure 3). 

#### 3.4.2. Sample No. 2

A 1-year-old male was diagnosed with an unspecified respiratory disorder in the pediatric intensive care unit of KAUH. The H1N1 strain of Influenza A (RNA virus) was detected with 1891 f. Additionally, *Moraxella catarrhalis, Staphylococcus aureus*, and *Streptococcus pneumoniae* were detected with frequencies of 535, 242, and 28, respectively. The Gram-negative bacteria *Escherichia coli* was found in the sample with 23 f. The maximum frequency among all pathogens in the sample was for the Gram-positive bacteria *Dolosigranulum pigrum* with 85,751 f, followed by the Gram-positive bacteria *Rothia mucilaginosa* (14,625 f), *Actinomyces graevenitzii* (12,585 f), and *Corynebacterium pseudodiphtheriticum* (2429 f), which have been reported to form part of the oropharyngeal flora in opportunistic human pathogen infections [14,15,16]. Three *Streptococcus* species that are primary inhabitants of the human upper respiratory tract and are also considered to be respiratory pathogens were found in the sample: *Streptococcus mitis* (758 f), *Streptococcus agalactiae* (81 f), and *Streptococcus pseudopneumoniae* (37 f) [17,18,19]. Further tests were done by the hospital on different samples from the patient, and Gram-positive cocci sepsis in the blood and ESBL *E. coli* from the respiratory sample culture were detected.

#### 3.4.3. Sample No. 3

The third throat swab sample belonged to an 84-year-old male with chronic renal failure who complained of productive cough and presented with widespread inspiratory and expiratory wheezing. *Candida albicans* was found in the sample with 2965 f in addition to pathogenic bacteria, which were *Haemophilus parainfluenzae* (16,865 f), *Escherichia coli* (588 f), and *Streptococcus pneumoniae* (230 f). In addition, the DNA virus Human gammaherpesvirus 4 (Epstein–Barr virus) was detected with (173 f). *Streptococcus mitis* (1275 f), *Streptococcus agalactiae* (282 f), and *Rothia mucilaginosa* (1257 f) were also detected in the sample. The maximum frequency (31,095) was found for the Gram-negative bacteria *Gemella haemolysans*. Other species from the same genus, *Gemella morbillorum* (2887 f) and *Gemella sanguinis* (4067 f), were detected in the sample. These three bacteria are known as normal microbiota of the mouth and have been reported as opportunistic pathogens that can cause some severe infections, which often occur in previously damaged tissue [20,21]. 

#### 3.4.4. Sample No. 4

A sample from a 1-year-old female diagnosed with a chest infection and endocarditis was analyzed. Sequence analysis of her throat swab showed two viruses: an RNA virus, Respiratory syncytial virus with 845 f, and a DNA virus, Human betaherpesvirus 5 (Cytomegalovirus) with 809 f. A number of pathogenic bacteria were also detected: *Streptococcus pneumoniae* (11 f), *Moraxella catarrhalis* (309 f), and *Dolosigranulum pigrum* with a maximum frequency of 12,087. *Neisseria meningitidi*, which is the most common non-neurological pathogen that causes pneumonia [22], was found in the sample with 10 f. In addition, *Streptococcus mitis* (1218 f), *Streptococcus pseudopneumoniae* (16 f), and *Corynebacterium pseudodiphtheriticum* (306 f) were found. 

#### 3.4.5. Sample No. 5

A sample from a 2-year-old female diagnosed with bronchopneumonia was tested. A number of pathogenic bacteria were found in her analyzed throat swab: *Streptococcus pneumoniae* (101 f), *Moraxella catarrhalis* (36 f), and *Dolosigranulum pigrum* with a maximum frequency 33,972. Several streptococcal species were detected: *Streptococcus mitis* (9294 f), *Streptococcus pseudopneumoniae* (388 f), *Streptococcus agalactiae* (140 f), and *Streptococcus anginosus* (5011 f), which are associated with pleuropulmonary infections [23]. Furthermore, *Corynebacterium pseudodiphtheriticum* was detected in the sample with 1396 f. In addition, sample analysis showed two viruses: an RNA virus, Respiratory syncytial virus (2398 f), and a DNA virus, Human adenovirus (41 with 45 f).

#### 3.4.6. Sample No. 6

Sequence analysis of a throat swab sample from a 12-year-old girl showed Influenza A virus H3N2 with 1365 f. *Haemophilus parainfluenzae* (2469 f) and *Escherichia coli* (4226 f) detected. In addition, a number of bacteria that have been reported to have pathogenic potential were found in the sample: *Granulicatella adiacens* (45,700 f) [24], *Capnocytophaga gingivalis* (26,919 f) [25], *Lautropia mirabilis* (74,316 f) [26], *Eikenella corrodens* (7923 f) [27], *Rothia mucilaginosa* (3622 f), *Peptoniphilus lacrimalis* (3520 f) [28], *Neisseria flavescens* (1617 f) [29], and *Actinomyces graevenitzii* (2546 f).

#### 3.4.7. Negative Control

A limited number of viruses were detected after CosmosID filtration in the negative control, which underwent the same extraction process, followed by the rest of the steps of the metagenomics protocol, without the depletion step (see Appendix B). They were identified as White clover cryptic virus 2, Red clover cryptic virus 2, Rosellinia necatrix partitivirus, Piscine myocarditis-like virus, and Dill cryptic virus. Only one pathogenic virus was found, Hepatitis C virus genotype 1, without being detected in any sample of the run. The detected frequencies of these microbes in the clinical samples did not exceed 1%, except in sample no. 1 with 5%. 

## 4. Discussion

Selection of the proper extraction protocol is a critical step for a successful clinical metagenomics procedure that serves to identify etiological agents that were not identified previously with the routinely available techniques. The challenge in this step is adapting an optimized protocol that is capable of extracting nucleic acids from diverse microbial taxa, varying from difficult-to-lyse organisms, such as yeasts, to organisms with easily degradable nucleic acids, such as RNA viruses. 

In the present study, we compared a number of extraction protocols to select the best method for nucleic acid isolation from a pool of pathogens with different pathogenic and genomic characteristics to include the major types of possible pathogens that might be found in a natural co-infected clinical sample. The mock sample used in this study include the following pathogens; ALKV, representing enveloped RNA viruses; AdV, representing nonenveloped DNA viruses; *S. aureus*, representing Gram-positive bacteria; *K. pneumoniae*, representing Gram-negative bacteria; and *C. neoformans*, representing encapsulated yeast. On the basis of these structural and genomic differences, each microorganism showed a different yield with each extraction method. Consequently, the identification of an ideal extraction protocol for all types of pathogens was a difficult task. Instead, the efficiency of the kits used in this study was estimated on the basis of the results outcome evaluated by highest number of reads for most of the included organisms, leading to a high coverage of the reference genome. 

The reads generated in this study showed that the best results were obtained for the extraction protocols that utilized bead beating in the lysis step. The first protocol was conducted using the PowerViral^®^ Environmental RNA/DNA Isolation Kit (MoBio samples), and the other protocol was the “Copurification” protocol of the ZymoBIOMICS™ DNA/RNA Miniprep Kit (Zymo samples). Although we used the approach of Leite et al. [30], who performed 2 cycles of 25-s agitation with a 5-s interval break, which they recommended to avoid possible degradation of nucleic acids and which was also reported by others [31,32], we improved the results of the MoBio and Zymo assays by performing the bead beating step at 4 °C and increasing the time to 5 min. Apart from the significant differences for the 5-min MoBio protocol compared with the 5-min Zymo protocol, the volume of the starting material for the MoBio extraction was 20% less than that for the Zymo kits (200 µL vs. 250 µL, respectively), and the number of steps of MoBio was fewer than that of Zymo, reducing the possibility of human error or contamination. 

Overall, all the extraction protocols showed ≈100% coverage for AdV and ALKV, regardless of the number of reads that achieved this coverage, which could be the result of their relatively short genomes of ≈35 kb and ≈10 kb, respectively, and the ease of extraction. 

Furthermore, the “Copurification” protocol of the ZymoBIOMICS™ DNA/RNA Miniprep Kit (Zymo samples) targeting both DNA and RNA was competitive with the parallel protocol for the same kit for extracting either DNA or RNA (ZRNA and ZDNA samples). There were no significant differences between the results of RNA sequencing, except for *S. aureus*, which had better mapped read results with ZRNA (*p* = 0.024) but better coverage with Zymo (*p* ≤ 0.001), or the results of DNA sequencing, except for the reads of *S. aureus* for Zymo (*p* = 0.049) and AdV for ZDNA (*p* ≤ 0.001). Similar results were obtained by Kresse et al. [33], who compared the separate and simultaneous protocols supplied with the truEXTRACT kit and found no preference for one over the other. Given the above data, our study showed that separate extraction did not necessarily lead to better results than the “Copurification” protocols when using the kit for clinical metagenomic investigations, making the “Copurification” protocol more suitable for clinical samples that are limited in quantity. 

Additionally, the reads produced from RNA sequencing using the KAPA RNA Hyper prep kit, which utilizes only RNA genomes for library preparation, were appropriate for the detection of all the pathogens used in the mock sample, even organisms with DNA genomes. This result supports the idea that infectious pathogens are transcriptionally active and that DNA pathogens can be identified by sequencing their rRNA [34,35]. Nonetheless, both libraries, DNA sequencing and RNA sequencing, are still recommended for the efficient coverage of all microbial taxa, as there was a significant difference in the DNA sequencing coverage of the bacteria used in the study (*p* = 0.001) compared with the coverage in RNA sequencing. Confidence in the results also increased when the same pathogen was detected in both libraries, as reported by Simner et al. [36].

The results from the clinical throat swabs showed that the chosen extraction method, which has the ability to extract DNA and RNA from different types of pathogens, avoids the use of more than one extraction method and consequently reduces the necessary quantity of the clinical sample. Moreover, it can help in constructing one protocol for clinical metagenomics, such as the one used in this study, targeting all taxa of etiological agents in the sample.

## 5. Conclusions

The above findings suggest that PowerViral^®^ Environmental RNA/DNA Isolation Kit with cooled 5-min bead beating in its lysis step can be useful for application in clinical metagenomics for samples with multiple infections from different taxa, whether RNA viruses or DNA microbes (fungus, bacteria, and DNA viruses), avoiding the need to use more than one extraction method. We recommend performing a large scale study using PowerViral^®^ Environmental RNA/DNA Isolation Kit to evaluate and validate the usefulness of this approach for clinical metagenomics application.

## Figures and Tables

**Table 1:** Genome sequences used for reference mapping in this study.

Microorganism	Reference Name	Accession
*C. neoformans*	*Cryptococcus neoformans* var. *neoformans* JEC21	AE017341-356
*K. pneumoniae*	*Klebsiella pneumoniae* subsp. *pneumoniae* HS11286	CP003223-228 + CP003200
*S. aureus*	*Staphylococcus aureus* subsp. *aureus* NCTC 8325	CP000253
AdV	Human mastadenovirus E strain HAdVE/USA_ New York/38813/2014/P4H4F4	KY996444
ALKV	Alkhumra hemorrhagic fever virus strain SCVHF001	JN860200

**Table 2:** Average reads per million (RPM) from RNA sequencing for the pathogens included in the mock sample using different protocols.

Average RPM	MagNA	Zol	MoBio	Zymo	ZRNA
*C. neoformans*	19,257	82,441	30,427	90,684	75,359
*K. pneumoniae*	784,302	117,122	255,302	184,597	177,745
*S. aureus*	46,263	182,489	210,541	143,569	216,660
AdV	11,193	21,834	54,898	53,243	7593
ALKV	2866	8182	2247	12,830	12,945

The highlighted cells contain the highest results without statistical significance between them.

**Table 3:** Average coverage percentage (coverage %) from RNA sequencing for the pathogens included in the mock sample using different protocols.

Average of Coverage %	MagNA	Zol	MoBio	Zymo	ZRNA
*C. neoformans*	0.04	0.06	0.03	0.09	0.08
*K. pneumoniae*	5.98	7.96	7.20	5.29	4.64
*S. aureus*	13.40	36.35	64.10	45.90	24.95
Adv	100.00	100.00	100.00	100.00	99.80
ALKV	100.00	100.00	100.00	100.00	100.00

The highlighted cells contain the highest results without statistical significance between them.

**Table 4:** Average reads per million (RPM) from DNA sequencing for the pathogens included in the mock sample using different protocols.

Average of RPM	MagNA	MoBio	Zymo	ZDNA
*C. neoformans*	34	601	56	163
*K. pneumoniae*	152,023	192,812	100,536	91,928
*S. aureus*	1590	40,813	37,205	34,785
Adv	119,851	53,233	40,054	72,556

The highlighted cells contain the highest results without statistical significance between them.

**Table 5:** Average coverage percentage (coverage %) from DNA sequencing for the pathogens included in the mock sample using different protocols.

Average of Coverage %	MagNA	MoBio	Zymo	ZDNA
*C. neoformans*	0.02	0.03	0.11	0.48
*K. pneumoniae*	13.51	12.41	13.51	13.51
*S. aureus*	19.90	23.20	99.85	99.90
Adv	100.00	100.00	100.00	100.00

The highlighted cells contain the highest results without statistical significance between them.

**Table 6:** The Averages of reads per million (RPM) and reference coverage percentage (coverage %) for the pathogens included in the mock sample with 5-min bead beating.

	Average of RPM		Average of Coverage %	
Sample Name	MoBio	Zymo	MoBio	Zymo
*C. neoformans*	1379	410	3.46	0.54
*K. pneumoniae*	232,653	240,061	18.57	18.45
*S. aureus*	51,102	42,645	99.90	99.80
Adv	145,277	80,502	100.00	100.00

The highlighted cells contain significant results.

**Table 7:** Heat map shows a comparison of MoBio protocol and Zymo protocol yields after 5-min bead beating based on the abundance of microbes detected from the CosmosID app.

Name	MoBio 5-min	Zymo 5-min
Naegleria fowleri		
*Staphylococcus aureus* subsp. *Aureus*		
*Staphylococcus lugdunensis*		
*Streptococcus*		
*Actinomyces* sp. HPA0247		
*Rothia*		
*Eikenella corrodens* ATCC 23834		
*Neisseria*		
*Kingella denitrificans* ATCC 33394		
*Pseudomonas aeruginosa*		
*Aggregatibacter segnis* ATCC 33393		
*Stenotrophomonas maltophilia*		
*Campylobacter*		
*Fusobacterium*		
*Porphyromonas*		
*Prevotella nanceiensis* DSM 19126		
Human metapneumovirus		
Pseudomonas phage		
Staphylococcus phage		
Staphylococcus prophage phiPV83		

The colors grade from the red representing the maximum score to green for the minimum score. The gray color represents no score.

## Appendix A

### Bead Beating Optimization

The optimization was performed on a mixture of 1:1 diluted *C. neoformans* and 1:10 diluted ALKV, representing the toughest structure and the more fragile genome among the spiked microorganisms used in this study; the microbes were diluted in phosphate-buffered saline (PBS) to 200 µL. By following the PowerViral^®^ Environmental RNA/DNA Isolation kit (MoBio protocol), different bead beating times were used as follows: sample 1 was beaten for 25 s, followed by a 5 s interval and another 25 s of agitation. Then, the beating times were increased consecutively from samples 2 to 11 with an interval of 5 s to reach a total time of 5 min in sample number 11. Finally, sample number 12 was beaten continuously for 5 min.

The results of different bead beating cycles were assessed by real-time PCR before proceeding to NGS. The primers and probes targeting *C. neoformans* and ALKV are listed in Table A1. The reaction was performed on an Applied Biosystems^®^ 7500 fast (Applied Biosystems, Singapore) using the QuantiFast-Probe-RT-PCR Kit (Qiagen, Hilden, Germany) and 5 µL of the extracted sample.

**Table A1:** Primers and probes used in this study to detect *C. neoformans* and ALKV.

Target	Name	Sequence	Ref
*C. neoformans*	CneoFwd	5′-GCCGCGACCTGCAAAG-3′	[37]
	CneoRev	5′-GGTAATCACCTTCCCACTAACACAT-3′	
	CneoProbe	5′-FAM-ACGTCGGCTCGCC-BHQ1-3′	
ALKV	KFDVF	5′-GAGGCTGCGTCATGGACAT-3′	[38]
	KFDVR	5′-CCTTGATGTTCGTGAGGGTGTT-3′	
	KFDVP	5′-HEX-CAACGTGGTTCAGGY^1^CAGGTGGT-BHQ1-3′	

1 This base was changed in this study from C to Y(C/T) to detect ALKV in addition to Kyasanur forest disease virus (KFDV), originally targeted by the probe.

The Ct of *C. neoformans* showed a gradual decrease with the increase in the beating period, from 26.74 for 2 × 25 s separated by a 5-s break to 22.7 with continuous 5-min agitation. ALKV showed a slight decrease in Ct values between the highest and lowest time intervals of bead beating used in this experiment (20.87 vs. 21.2) (Table A2).

**Table A2:** The results of the real-time PCR cycle threshold (Ct) for different cycles of bead beating.

Name	Bead Beating Cycles	Total Beating	*C. neoformans* Ct	ALKV Ct
Sample 1	25 s.5 s × 2	50 s	26.74	20.87
Sample 2	25 s.5 s × 3	1 min + 15 s	26.28	21.25
Sample 3	25 s.5 s × 4	1 min + 40 s	25.69	21.46
Sample 4	25 s.5 s × 5	2 min + 5 s	24.95	21.66
Sample 5	25 s.5 s × 6	2 min + 30 s	23.71	21.37
Sample 6	25 s.5 s × 7	2 min + 55 s	25.12	21.93
Sample 7	25 s.5 s × 8	3 min + 20 s	25.29	21.68
Sample 8	25 s.5 s × 9	3 min + 45 s	25.24	20.65
Sample 9	25 s.5 s ×10	4 min + 10 s	23.95	21.61
Sample 10	25 s.5 s × 11	4 min + 35 s	24.6	22.68
Sample 11	25 s.5 s × 12	5 min	23.82	20.72
Sample 12	5 min	5 min	22.7	21.2

## Appendix B

### Metagenomics Protocol

An establishment protocol was followed that targeted all microbes in a sample, disregarding their taxa or genome type. After extraction using the maximum recommended elution volume (100 μL), a concentration step using RNA Clean & Concentrator™-5 (Zymo Research, Irvine, CA, USA) was applied following the kit instructions to 12 μL. Complementary DNA synthesis was performed using 10 μL of the concentrated yield and mixed with 10 µM of nonribosomal primers, consisting of 96 hexanucleotides chosen by Endoh et al. [39] to convert only RNA viruses to cDNA, leading to rRNA depletion. The mixture was heated to 65 °C for 5 min and incubated on ice for 5 min. Then, 1x of first-strand buffer, 0.1 M of dithiothreitol (DTT), 40 U of RNase OUT (Invitrogen, Carlsbad, CA, USA) and 400 U of SuperScript III Reverse Transcriptase (Invitrogen, Carlsbad, CA, USA) were added to the denatured RNA. The mixture was incubated at 25 °C for 5 min and then at 55 °C for 60 min, followed by enzyme inactivation by increasing the temperature to 70 °C for 15 min. Double-strand DNA synthesis was carried out using Polymerase I, Large (Klenow) Fragment (NEB, Ipswich, MA, USA), by preparing 1x NEBuffer 2, 10 mM of dNTPs, 10 U of Klenow and 20 U of Rnase H (NEB, Ipswich, MA, USA). This mixture was added to the previous reaction and incubated at 37 °C for 60 min, followed by holding at 4 °C. The reaction product was then purified using 1.8x of Agencourt AMPure XP beads (Beckman Coulter, Brea, CA, USA) following the kit instructions. NEBNext Microbiome DNA Enrichment Kit (NEB, Ipswich, MA, USA) was only applied in samples no. 3, 4, and 5 following its instructions to deplete human DNA, followed by a final purification step using DNA Clean & Concentrator (Zymo Research, Irvine, CA, USA) and elution in 35 μL. All purified samples, with or without the depletion step, were subject to library preparation using Nextera DNA Flex Library Prep as described previously.

## Appendix D

**Table A3:** Heat map for the filtered microbes in the studied clinical samples based on frequencies generated from the CosmosID app.

	Sample Number	1	2	3	4	5	6
Fungi	*Candida albicans*	387	0	2965	0	0	0
	*Candida glabrata*	799	0	0	0	0	0
	*Kluyveromyces marxianus*	352	0	0	0	0	0
Bacteria	*Abiotrophia defectiva*	0	0	0	0	0	5591
	*Achromobacter*	1690	0	0	0	0	0
	*Actinomyces graevenitzii*	0	12,585	0	0	0	2546
	*Actinomyces odontolyticus*	0	0	0	0	0	1647
	*Aggregatibacter*	116	0	0	0	0	0
	*Atopobium parvulum*	0	0	0	0	0	8819
	*Atopobium sp ICM42b*	0	0	0	0	0	9724
	*Atopobium sp ICM58*	0	0	0	0	0	94,149
	*Bacteroidales Order 27*	0	0	0	0	0	869
	*Bifidobacterium breve*	0	141	0	0	0	0
	*Campylobacter concisus*	0	0	744	0	0	0
	*Campylobacter showae*	0	0	286	0	0	0
	*Candidate division TM7*	0	0	3017	0	0	0
	*Capnocytophaga*	0	0	378	0	0	0
	*Capnocytophaga gingivalis*	0	0	0	0	0	26,919
	*Capnocytophaga ochracea*	0	0	613	0	0	0
	*Capnocytophaga sp 123 Branch*	0	0	0	0	0	383
	*Capnocytophaga sp. Oral*	0	0	661	0	0	0
	*Christensenella Family Node 1*	0	0	0	0	0	1381
	*Corynebacterium matruchotii*	0	0	1739	0	0	0
	*Corynebacterium pseudodiphtheriticum*	0	2429	0	306	1396	0
	*Dermabacter*	0	1562	0	0	0	0
	*Dolosigranulum pigrum*	0	85,751	0	12,087	33,972	0
	*Eikenella corrodens*	0	0	0	0	0	7923
	*Escherichia coli*	0	23	588	0	0	4226
	*Fusobacterium nucleatum*	0	0	445	0	0	0
	*Fusobacterium periodonticum*	0	0	122	0	0	1283
	*Gemella*	0	8	0	19	18	0
	*Gemella haemolysans*	0	0	31,095	0	0	0
	*Gemella morbillorum*	0	0	2887	0	0	0
	*Gemella sanguinis*	0	0	4067	0	0	0
	*Granulicatella*	0	78	44	0	0	0
	*Granulicatella adiacens*	0	0	0	0	0	45,700
	*Haemophilus*	0	10	0	26	0	0
	*Haemophilus haemolyticus*	0	0	0	25	0	0
	*Haemophilus influenzae*	398	0	0	0	0	0
	*Haemophilus parainfluenzae*	0	0	16,865	0	0	2469
	*Kingella denitrificans*	0	0	3037	0	0	0
	*Lachnoanaerobaculum saburreum*	0	0	1223	0	0	906
	*Lachnoanaerobaculum sp ICM7*	0	0	0	0	0	111,698
	*Lachnospiraceae*	0	0	30	0	0	0
	*Lachnospiraceae bacterium oral*	0	0	0	0	0	8288
	*Lachnospiraceae oral*	0	0	0	0	0	3738
	*Lactobacillus*	0	50	0	0	0	0
	*Lactobacillus fermentum*	0	8	0	0	0	0
	*Lactobacillus gasseri*	0	123	0	0	0	0
	*Lactobacillus oris*	0	0	0	0	1888	0
	*Lactobacillus salivarius*	0	0	0	0	24,443	0
	*Lactobacillus vaginalis*	0	0	0	0	56,698	0
	*Lautropia mirabilis*	0	0	0	0	0	74,316
	*Leptotrichia*	0	80	0	0	0	0
	*Leptotrichia buccalis*	0	0	3077	0	0	0
	*Leptotrichia hofstadii*	0	0	1694	0	0	0
	*Leptotrichia shahii*	0	0	988	0	0	0
	*Leptotrichia sp. Oral*	0	0	5321	0	0	56,298
	*Leptotrichia trevisanii*	0	0	1530	0	0	0
	*Leptotrichia wadei*	0	0	1497	0	0	0
	*Moraxella catarrhalis*	867	535	0	309	36	0
	*Negativicoccus succinicivorans*	0	0	0	0	0	2945
	*Neisseria cinerea*	0	0	0	1518	0	0
	*Neisseria flavescens*	0	0	0	0	0	1617
	*Neisseria lactamica*	0	0	0	1260	0	0
	*Neisseria meningitidis*	0	0	0	10	0	0
	*Neisseria mucosa*	0	0	0	0	0	1856
	*Neisseria polysaccharea*	0	0	0	817	0	0
	*Neisseria subflava*	0	0	0	0	0	3363
	*Oribacterium*	0	0	49	0	0	0
	*Oribacterium 27619 Branch*	0	0	0	0	0	835
	*Oribacterium parvum*	0	0	0	0	0	690
	*Oribacterium sinus*	0	0	5749	0	0	8051
	*Peptoniphilus lacrimalis*	0	0	0	0	0	3520
	*Peptostreptococcus 26809 Branch*	0	0	0	0	0	25
	*Porphyromonas*	11	110	0	0	0	0
	*Porphyromonas sp COT 1360 Branch*	0	0	0	0	0	88
	*Porphyromonas sp oral*	0	0	0	0	0	133,736
	*Prevotella*	170	0	8	0	54	0
	*Prevotella denticola*	0	0	26	0	0	0
	*Prevotella histicola*	0	2166	1799	0	0	184,392
	*Prevotella intermedia*	0	0	476	0	0	0
	*Prevotella melaninogenica*	0	0	11,664	0	0	113,376
	*Prevotella nanceiensis*	0	0	0	0	0	131,564
	*Prevotella nigrescens*	0	0	820	0	0	174
	*Prevotella oris 764 Branch*	0	0	0	0	0	381
	*Prevotella pallens*	0	0	4867	0	0	87,205
	*Prevotella salivae*	0	0	1358	0	0	61,527
	*Prevotella scopos*	0	0	0	0	0	18,536
	*Prevotella shahii ]*	0	0	0	0	0	120,417
	*Prevotella sp. ICM33*	0	0	14,193	0	0	112,154
	*Prevotella veroralis*	0	0	1078	0	0	0
	*Propionibacteriaceae*	0	0	0	0	12	0
	*Propionibacterium acnes*	0	0	0	0	0	420
	*Propionibacterium sp DORA 15*	0	0	0	0	0	1892
	*Rothia dentocariosa*	0	0	1728	0	0	0
	*Rothia mucilaginosa*	0	14,625	790	0	0	3622
	*Solobacterium moorei*	0	0	0	0	0	731
	*Staphylococcus aureus*	0	242	0	0	0	0
	*Staphylococcus epidermidis*	0	0	0	0	0	48
	*Staphylococcus sp DORA 6 22*	0	0	0	0	0	247
	*Stomatobaculum longum*	0	0	0	0	0	27,025
	*Streptococcus*	47	15	241	0	2368	0
	*Streptococcus agalactiae*	0	81	282	0	140	0
	*Streptococcus anginosus*	0	0	0	0	5011	0
	*Streptococcus australis*	0	0	0	0	0	32,772
	*Streptococcus infantis*	0	0	0	0	0	9143
	*Streptococcus mitis*	0	758	1257	1218	9294	0
	*Streptococcus oralis*	0	0	1671	0	0	0
	*Streptococcus parasanguinis*	0	0	0	0	0	3898
	*Streptococcus peroris*	0	0	0	0	0	1559
	*Streptococcus pneumoniae*	0	28	230	11	101	0
	*Streptococcus pseudopneumoniae*	0	37	0	16	388	0
	*Streptococcus salivarius*	0	1966	0	0	9599	0
	*Streptococcus salivarius*	0	0	0	0	0	0
	*Streptococcus sp I P16*	0	0	0	0	0	3220
	*Streptococcus sp. C150*	0	2308	0	0	20,769	0
	*Streptococcus sp. C150*	0	0	0	0	0	0
	*Streptococcus sp. DBCMS*	0	0	2063	0	0	0
	*Streptococcus thermophilus*	0	0	0	0	10	0
	*Streptococcus vestibularis*	0	0	0	0	3396	0
	*Tannerella sp oral*	0	0	0	0	0	84
	*Veillonella*	0	101	49	0	144	0
	*Veillonella atypica*	0	520	0	0	0	2196
	*Veillonella dispar*	0	0	6874	0	0	138,748
	*Veillonella parvula*	0	0	543	0	0	3730
	*Veillonella sp oral*	0	0	0	0	0	327,939
	*Veillonella sp. 3_1_44*	0	0	355	0	0	0
	*Veillonella sp. HPA0037*	0	837	0	0	0	0
Viruses	Dill cryptic virus	158	158	64	22	203	211
	Dulcamara mottle virus	0	0	0	0	0	12
	Groundnut ringspot and Tomato chlorotic spot virus reassortant	0	0	20	0	0	0
	Haemophilus virus	0	0	0	0	0	68
	Hp1virus	0	13	115	24	0	0
	Human adenovirus 41	0	0	0	0	45	0
	Human betaherpesvirus 5 (CMV)	0	0	0	809	0	0
	Human gammaherpesvirus 4 (EBV)	0	0	173	0	0	0
	Human orthopneumovirus (RSV)	0	0	0	845	2398	0
	Human parainfluenza virus 2	2363	0	0	0	0	0
	Influenza A virus (H1N1)	0	1891	0	0	0	0
	Influenza A virus (H3N2)	0	0	0	0	0	1365
	Piscine myocarditis-like virus	0	31	0	7	52	106
	Pseudomonas virus Pf1	65	0	0	0	0	0
	Red clover cryptic virus	137	104	45	26	146	220
	Rosellinia necatrix partitivirus	82	95	85	66	261	311
	Sclerotinia sclerotiorum partitivirus	0	0	0	0	0	13
	Staphylococcus virus	21	67	0	0	0	27
	Streptococcus virus	0	0	0	0	720	0
	Tobacco mosaic virus	0	0	0	0	27	0
	Torque teno mini virus	0	0	0	10	0	0
	Vicia cryptic virus	46	31	0	0	82	0
	White clover cryptic virus	18	0	16	6	0	193

1 The taxonomy is at the strain level if found. If there were different sub-strains, all frequencies were added to its strain. 2 The blue-colored microbes are the ones found in the negative control.
